# Endoscopic vacuum therapy achieves complete resolution of anastomotic leakage after transanal total mesorectal excision in low rectal cancer: a full therapeutic course report

**DOI:** 10.1055/a-2742-5701

**Published:** 2025-11-28

**Authors:** Mi Tang, Huanyu Hu, Jiaming Liao, Botao Qian, Yunhong Tian

**Affiliations:** 1 556508 Department of General Surgery, The Affiliated Hospital of Southwest Medical University, Luzhou, China; 2 662481 Department of General Surgery, Nanchong Central Hospital Affiliated to North Sichuan Medical College, Nanchong, China

Anastomotic leakage remains one of the most serious and challenging complications following rectal cancer surgery 1 2 . Recently, our team found that endoscopic vacuum therapy (EVT) can improve the healing rate of anastomotic fistulas after rectal cancer surgery and shorten the overall treatment duration 3 . However, to date, no video in the current e-video database has comprehensively documented the complete healing process of anastomotic leakage following curative-intent rectal cancer surgery 4 . Here, we present a case of anastomotic leakage successfully treated with EVT after transanal total mesorectal excision (taTME) for low rectal cancer.

This case involves a 69-year-old man diagnosed with rectal cancer (pathological stage pT3N0M0), who underwent taTME with a protective ileostomy. Anastomotic leakage was diagnosed on postoperative day 21.

A total of five EVT sessions were performed, with an interval of 5–6 days between each treatment ( Video 1 ). The total treatment duration was 28 days. Colonoscopy revealed an anastomotic leak located 4.7 cm above the anal verge ( Fig. 1 ). A handmade EVT device was positioned within the pus cavity through the anastomotic defect ( Fig. 2 ). On the fifth day after the initial EVT session, new granulation tissue was observed at the anastomotic defect. Eighteen days after the initial EVT treatment ( Fig. 3 ), the defect had significantly decreased in size, with continued proliferation of granulation tissue. One week after completing all treatments, colonoscopy was performed for follow-up ( Fig. 4 ), revealing complete healing of the anastomotic leak. Two months after treatment completion ( Fig. 5 ), barium enema radiography showed no leakage or stenosis, confirming a well-healed anastomosis.

Endoscopic vacuum therapy is performed to close a rectal anastomotic leakage.Video 1

**Fig. 1:**
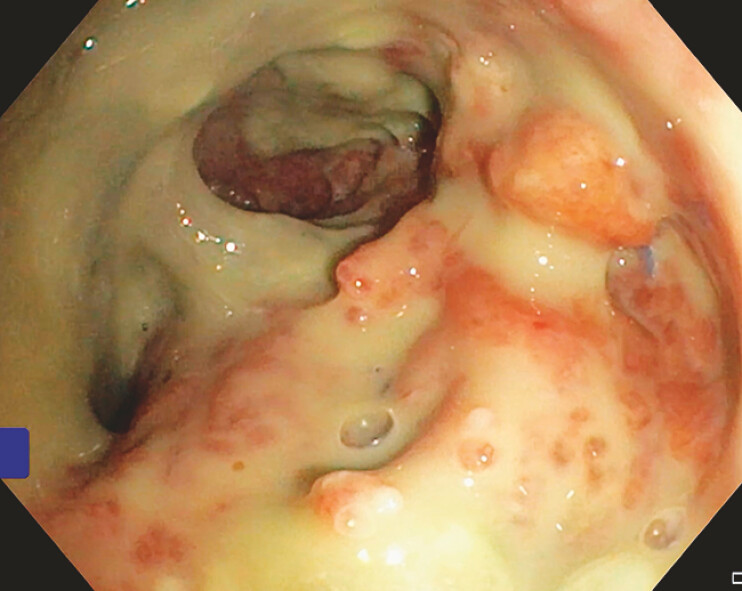
Colonoscopy revealed anastomotic leakage.

**Fig. 2:**
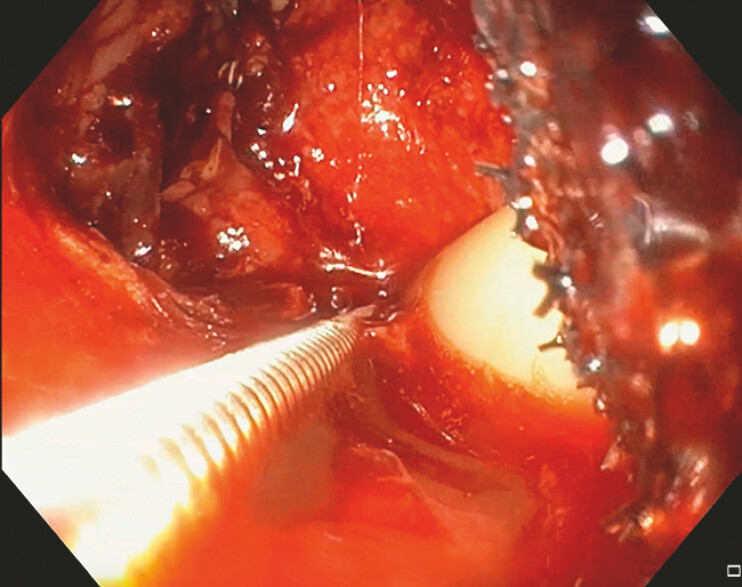
Place the EVT device. EVT, endoscopic vacuum therapy.

**Fig. 3:**
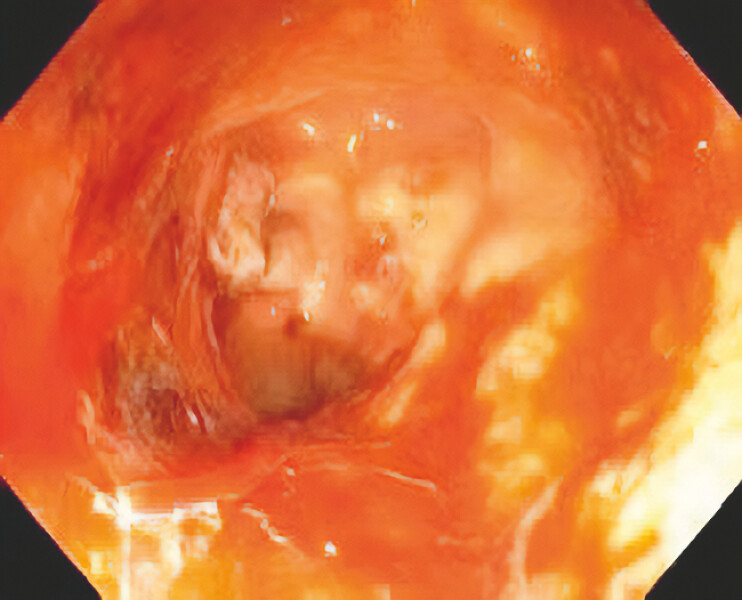
The anastomotic leakage was significantly reduced.

**Fig. 4:**
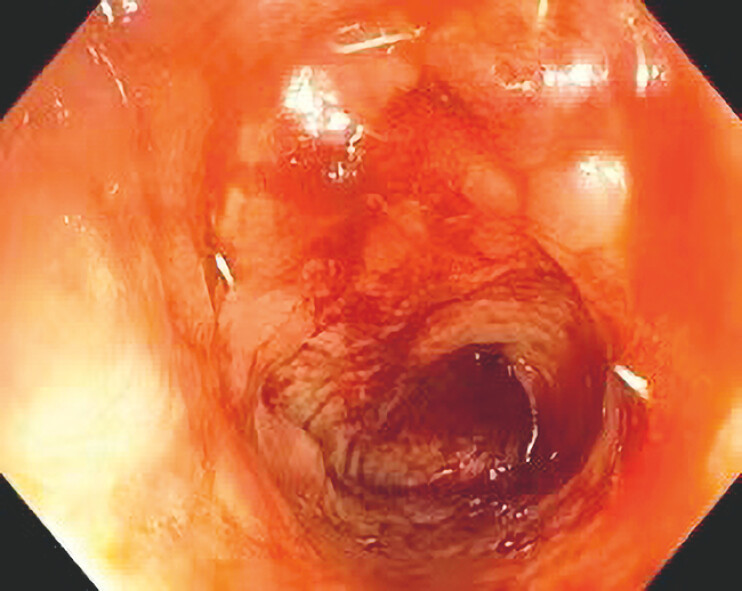
Colonoscopy was performed after the treatment.

**Fig. 5:**
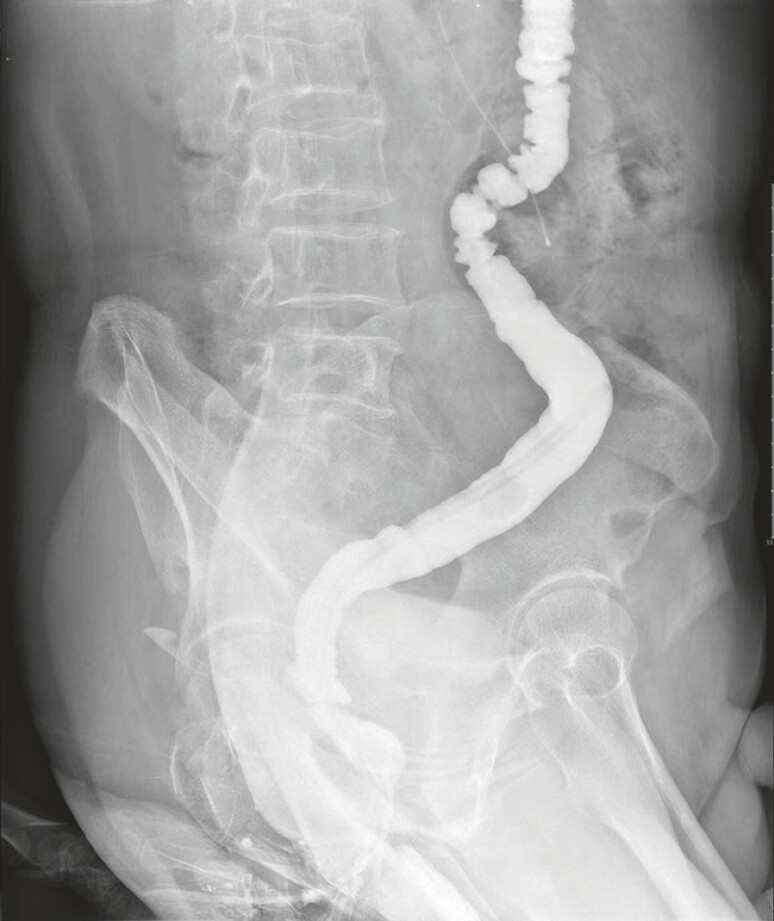
Postoperative barium enema.

To the best of our knowledge, this is the first report describing the complete treatment process of EVT for rectal anastomotic leakage, offering a useful reference for the wider application of EVT in treating anastomotic leakage after low rectal cancer surgery.

Endoscopy_UCTN_Code_TTT_1AQ_2AG

## Notes

Endoscopy E-Videos https://eref.thieme.de/e-videos
*E-Videos* is an open access online section of the journal *Endoscopy* , reporting on interesting cases and new techniques in gastroenterological endoscopy. All papers include a high-quality video and are published with a Creative Commons CC-BY license. Endoscopy E-Videos qualify for HINARI discounts and waivers and eligibility is automatically checked during the submission process. We grant 100% waivers to articles whose corresponding authors are based in Group A countries and 50% waivers to those who are based in Group B countries as classified by Research4Life (see: https://www.research4life.org/access/eligibility/ ). This section has its own submission website at https://mc.manuscriptcentral.com/e-videos .
